# PROTOCOL: A systematic review and meta‐analysis of randomised controlled trials evaluating the impact of parenting programmes for parents of adolescents (10–18 years) on adolescent mental health outcomes, positive development and the parent–adolescent relationship

**DOI:** 10.1002/cl2.1146

**Published:** 2021-02-07

**Authors:** Kylie Burke, Cassandra K. Dittman, Elana J. Forbes, Elizabeth Eggins

**Affiliations:** ^1^ Parenting and Family Support Centre, School of Psychology The University of Queensland Brisbane Queensland Australia; ^2^ School of Health, Medical and Applied Sciences Central Queensland University Bundaberg Queensland Australia; ^3^ School of Social Science, The University of Queensland Brisbane Queensland Australia

## 1 BACKGROUND FOR THE REVIEW

### 1.1 The problem, condition or issue

Mental health problems among adolescents are highly prevalent across the world, with international rates ranging from 10% to 20% (Collishaw, 2015; Kieling et al., 2011). In Australia, the 12‐month prevalence of mental disorders has been estimated at 13.9% with the most common disorders being behavioural (e.g., attention‐deficit/hyperactivity disorder and conduct) and anxiety disorders (Lawrence et al., 2016). Similar overall and disorder‐specific prevalence rates have been reported in the United States (Ghandour et al., 2019), the UK and Europe (Collishaw, 2015), with data indicating stability in prevalence over time (Ghandour et al., 2019; Lawrence et al., 2016). Clinical diagnosis and treatment of mental health disorders in adolescents has also been shown to be increasing over recent decades (Collishaw, 2015) indicating significant burden on individuals, families, communities and health systems.

Adolescent mental health problems have far‐reaching and potentially long‐lasting negative effects on the adolescents themselves, their families, and the wider community. Such adolescents are more likely to become socially and academically disengaged (McGue & Iacono, 2005; Smart et al., 2005; Smart, 2008), engage in criminal behaviour (Aebi et al., 2014; Bosick et al., 2015; Hughes et al., 2020), and have ongoing mental health, relationship and employment challenges in adulthood (Copeland et al., 2009, 2014; Hale et al., 2015; Naicker et al., 2013; Patton et al., 2014). Adolescents have also been demonstrated to engage in alcohol and substance use at relatively high levels (Guerin & White, 2020; Swendsen et al., 2012). Substance use and mental health problems such as depression have a bi‐directional relationship with significant implications for adolescent wellbeing and transition to adulthood (Isaksson et al., 2020; Liu et al., 2018). Further, Sellers et al (Sellers et al., 2019) in their study of cross‐time changes in outcomes following mental health problems during childhood using three UK longitudinal cohort studies and covering four decades of child mental health prevalence data, demonstrated that the experience of mental health issues during childhood has become more strongly associated with social, educational and mental health problems over time. Arguably, current rates of adolescent psychological, social, and educational issues indicate that many adolescents worldwide are “slipping through the cracks” and not being well‐served by the delivery of prevention, early intervention or treatment programmes.

Reducing the prevalence of problems among adolescents is only one part of addressing life‐long risk and poor outcomes. It is equally as important to promote the social, psychological and self‐regulatory skills and attributes that allow adolescents to become successful learners, build healthy relationships and to become healthy and productive contributors to their families and communities. Multiple studies and reviews of positive development have identified a range of psychological and emotional characteristics that promote positive mental health and social development in young people, including self‐regulation and self‐esteem, coping and persistence, responsibility and decision‐making, problem solving, motivation and achievement, having a future orientation; and, connectedness to peers, family and community and to institutions such as educational facilities (Arnett, 2000; Ciocanel et al., 2017; Curran & Wexler, 2017; García‐Poole et al., 2019; Lerner et al., 2009; O'Connell et al., 2009; Sardiñas et al., 2017).

While the call for greater focus on health prevention and promotion has increased in recent years, there is still a relative lag in the application of this work to adolescence in comparison to adults (Van Allen et al., 2017). Much of the literature examining interventions for adolescents has focused on prevention and treatment programmes targeted directly to adolescents with an emphasis on reducing or treating mental health and social problems. While systematic reviews have indicated evidence for interventions targeting adolescents with anxiety (James et al., 2015), systematic reviews of others, such as depression (Cox et al., 2014), social skills training for adolescents with ADHD (Storebø et al., 2019) or school‐based interventions for substance disorder (Carney et al., 2016) have shown mixed effects or inadequate ability to assess outcomes due to low quality studies. Prevention effects are also unclear. For example, Hetrick et al. (2016) Cochrane systematic review of the effects of cognitive behavioural therapy (CBT), third‐wave CBT and interpersonal therapy for preventing depression found only modest effects for short‐term positive effects with targeted populations (i.e., those with depression symptoms), and no effects for interventions delivered to universal populations. Exploration of the effects of these programmes is also complicated by low quality research designs. There is also a lack of good quality studies exploring the effects of interventions on adolescent positive development (Ciocanel et al., 2017). Thus, while directly targeting adolescents offers benefits for some conditions and at some population levels (treatment and targeted prevention), other types and targets of intervention are also important to consider.

The family context and parenting are two key targets for intervention. There is considerable evidence to suggest that harsh, ineffective parenting and/or poor‐quality relationships between parents and children are important precursors for mental health problems in children and adolescents (Odgers et al., 2008; Yap et al., 2014), with difficulties in childhood and adolescence translating into more serious difficulties in adulthood. Adolescents who have poor quality relationships with their parents are more likely to become socially and academically disengaged, engage in criminal behaviour (Farrington et al., 2009), and have ongoing relationship and employment challenges in adulthood (Hale et al., 2015). Conversely, a close parent–adolescent relationship and parenting characterised by effective conflict management, clear communication of expectations and rules, and appropriate limit setting and monitoring, is associated with important social and academic competencies in adolescence, including academic engagement and achievement (Kelly et al., 2012), capacity to manage behaviour and emotions (Farley & Kim‐Spoon, 2014), and better social and community connectedness (Smart et al., 2008). Furthermore, effective parenting has been shown to be a key factor protecting against a range of negative adolescent outcomes including truancy and other externalising behaviour difficulties (Wang et al., 2011), early sexual experience and alcohol and other drug use (Kelly et al., 2011).

However, despite the known role of parenting and other family factors in child and adolescent mental health problems, there has been a lack of synthesis of the evidence for interventions that involve parenting for adolescents. Decades of carefully conducted trials have demonstrated the efficacy of parenting programmes based on social learning theory in reducing difficult child behaviours and increasing adaptive child behaviours, parent competence and wellbeing (e.g., Dretzke et al., 2009; Eyberg et al., 2008; van Aar et al., 2017) and preventing antisocial behaviour and delinquency (Piquero et al., 2016). Yet, in spite of the strength of the evidence for the continuing importance of parenting in adolescence, the parenting field has focused almost exclusively on preventing problems of childhood and adolescence by working with parents of preadolescent children (Baumel et al., 2016; Chu et al., 2012; Spencer et al., 2020; Thomas & Zimmer‐Gembeck, 2007; Webster‐Stratton & Taylor, 2001). This lack of attention directed towards developing programmes for parents of adolescents is likely due to the pre‐eminence of early childhood as a critical intervention point, in conjunction with a belief that parental influence diminishes over time as adolescent behaviour becomes increasingly individually determined and peer influence strengthens (Kazdin, 2008). Given the significant and persistent challenges associated with mental health problems in adolescents, a clear understanding of the role and/or potential of parenting interventions with this age group is needed. Thus, it is essential to explore the evidence relating to the availability and effectiveness of parenting programmes that specifically target the adolescent years.

### 1.2 The intervention

This review will include any parenting intervention that aims to promote parenting practices and the parent–adolescent relationship during the developmental period from age 10–18 years. It is expected that included interventions will cover a range of theoretical paradigms and approaches, including but not limited to parenting interventions based on behavioural principles and social learning theory, attachment theory (including Interpersonal therapy), family systems theories, relational frame theory/acceptance and commitment therapy and nonviolent resistance theory. Thus, it is expected that programmes such as the Teen Triple P program (Ralph & Sanders, 2004), the Strengthening Families Program (Kumpfer & Magalhães, 2018) and the Nonviolent Resistance training model (Weinblatt & Omer, 2008) will be identified and considered for inclusion.

Parenting interventions have relevance and applications across the continuum from health promotion to prevention, early intervention and the treatment of acute and chronic serious mental health disorders in young people. Parents are tasked with the ongoing responsibility of helping their child to develop the skills they need to be successful adults. Whether in the absence or presence of mental illness and other potential adverse childhood experiences, this includes providing a safe, warm and loving environment characterised by clear boundaries and effective supervision that enables the young person to grow, learn, fail and succeed. When mental health disorders are present, parents are also critical for helping their child to manage their symptoms and maintain their treatment plans (Burke, 2017).

Therefore, to produce a comprehensive review of the extant evidence, we will use a relatively broad inclusion threshold for study inclusion. Specifically, the review will include studies where the intervention takes a prevention, early intervention or treatment focus (Select Committee on Mental Health, 2006). Preventive interventions are implemented prior to the initial onset of a disorder with the objective of preventing the development of the disorder by reducing risk factors and enhancing any protective factors associated with the targeted issue. Prevention interventions can be implemented at a universal (targeting the health of the whole population) or selected (targeting at‐risk populations, e.g., targeting parenting programmes specifically to teenage parents) level. Early intervention programmes are implemented with individuals showing early indicators or symptoms of problems and in the context of mental health disorders, during a first episode. The aim of early intervention is to prevent the progression of issues into diagnosable disorders or other serious and long‐lasting issues and to reduce the impact of any early indicators. Treatment interventions are designed to reduce, control or remediate symptoms and where possible underlying causes of issues. Further, they aim to reduce and manage the short‐ and long‐term impacts of the consequences of mental health and associated concerns.

We acknowledge that parenting programmes targeting adolescence are likely to overlap with programmes that predominantly target the adolescent directly and that contain psychoeducation components for parents. However, for the purposes of this review we specifically define parenting interventions as those that have a stated aim to improve parenting practices and/or the parent–adolescent relationship via direct engagement with parents. While interventions may include adolescent or broader family components the intervention synthesised in the final review must have a substantive parenting component that is of equal or greater dosage than any other programme component. To comprehensively synthesise the extant evaluation literature, this review will include studies where the intervention (manualised or nonmanualised) are delivered in any modality (e.g., face‐to‐face, group‐based, individual, telehealth). Similarly, targets of the intervention may vary, providing that the parent/carer is defined as a primary rather than secondary target. Intervention targets may include: parent only, parent–adolescent dyads/triads, family‐based interventions or multiple systems approaches (e.g., family and school).

### 1.3 How the intervention might work

Adolescence is a developmental period that is characterised by significant psychological, social and physical changes that result in attainment of greater autonomy, identity and importance of relationships outside the family (Steinberg, 2017). There are a number of possible mechanisms by which parenting interventions might work in this context. Some of these are likely to be consistent with the mechanisms by which evidence‐based parenting interventions work with younger children. This evidence suggests that parenting interventions using learning processes such as behavioural rehearsal (information provision, modelling, rehearsal of new skills and provision of feedback) to enhance parents' knowledge and skills result in large effects on parental behaviour and child outcomes in younger children (Wyatt Kaminski et al., 2008). These strategies increase parents understanding, confidence and capacity to implement strategies that enhance their communication and relationship with their children and as such it is likely that parenting interventions for parents of adolescents will work via similar mechanisms to encourage appropriate behaviour while assisting parents to manage more difficult issues such as risk taking. Research has for example shown that parenting interventions that target strategies such as the provision of emotional support, and the development of parenting skills that improve the relationship with the child in ways that support positive behaviour and offer strategies to deal with negative or challenging behaviours result in positive outcomes for children (Sanders et al., 2014), with the more limited evidence also demonstrating positive outcomes for adolescents (Medlow et al., 2016).

### 1.4 Why it is important to do this review

Serious mental illness is common in children and adolescents worldwide and is associated with significant and wide‐ranging functional impairments that continue into adulthood (Collishaw, 2015). Thus, prevention and treatment of mental health problems in children is a major international challenge. To effectively address this challenge we require more effective and evidence‐based, contextually driven approaches to reducing symptoms and promoting factors associated with well‐being (Burke, 2017). Parenting and the parent–adolescent relationship is a critical aspect of prevention, treatment and recovery for adolescents. In a prevention and early intervention context, parents have the task of supporting, teaching and managing their adolescents as they move towards defining their social identity and take on increased autonomy and responsibility. In treatment contexts, the parental role extends to assisting their adolescent to obtain support, to manage their symptoms and to promote activities and behaviours associated with wellbeing (Burke, 2017).

This systematic review has direct relevance to policy within the authors' country of residence (Australia) and internationally. Child and adolescent health and/or mental health policies around the world (e.g., the WHO's Global Accelerated Action Plan for the Health of Adolescents and Global Strategy for Women's, Children's and Adolescent's Health (2016–2030); the UK's National Health Services' Long Term Plan; the U.S. Department of Health and Human Services Healthy People agenda) emphasise the importance of the adolescent period as a critical transition point in the prevention and treatment of mental health problems and the promotion of health and wellbeing, along with the importance of evidence‐based interventions and the influential role. For example, this review will provide evidence‐based information for the action of “enhancing and promoting resources and mechanisms to support parenting in the middle years and adolescence,” which was identified as a neglected and less‐resourced area but still an influential period of transition where protective parenting has a strong impact on outcomes; and, for “tackling mental health and risky behaviours, such as building social and emotional coping skills in the middle years and during the transitions into adolescence and early adulthood” within the recently released Australian Government's National Action Plan for the Health of Children and Young People (2020–2030). The findings from this review will this support action within identified priority areas for governments across the world.

Despite the importance of parents and parenting in the protection and risk for the development of mental health problems in children and adolescents (Wang et al., 2011) as well as the large evidence base for parenting interventions with younger children, little attention has been given to synthesising the evidence for these interventions for the adolescent years. For example, a meta‐analysis evaluating the effectiveness of child and adolescent psychotherapies identified only 13 studies in which parent or family focused interventions were evaluated (Weisz et al., 2013). This study aimed to determine if evidence‐based psychotherapies (EBPs), produced better outcomes that usual care in youth psychotherapy. EBPs were broadly defined as “any treatment listed in at least 1 of the published reviews systematically identifying EBPs for youths based on the level of empirical support” (Weisz et al., 2013, p. 751) and covering age range of 3–18 years. The theoretical approach and target (e.g., parent or adolescent) for the intervention were not specified. Further, while interventions that included a parenting component were included, this was not a specific focus and outcomes from parent‐included interventions were only briefly examined. The proposed study extends and updates this work in several ways: (1) it specifically targets the role of parenting interventions for the adolescent developmental period; and (2) broadens the search strategy to include a larger range of databases and grey literature with an updated search period.

A systematic review of available programmes for parents of adolescents and their impact on adolescent mental health outcomes is therefore needed to assess not only availability of evidence‐based parenting interventions for this cohort across prevention, early intervention and treatment contexts, but also the efficacy of parenting interventions for this cohort and their potential to contribute to the reduction of mental illness and promotion of skills associated with positive development in adolescents. There are several systematic reviews available that touch on parenting interventions for parents of adolescents. However, these have tended to be of narrow focus, such as targeting adolescent substance abuse (e.g., Allen et al., 2016; Kuntsche & Kuntsche, 2016); externalising mental health disorders (e.g., conduct disorder, antisocial behaviour; McCart et al., 2006; Medlow et al., 2016; Woolfenden et al., 2009) or internalising mental health problems (e.g., anxiety disorders, depression; Das et al., 2016; Gillham et al., 2000), or they are outdated or target a wider age range without a specific focus on adolescence. Others have targeted adolescents with existing diagnoses or clinical symptoms, meaning that the preventative role of parenting programmes is largely unexplored (e.g., Woolfenden et al., 2001). One other review of family‐based interventions for child and adolescent mental health disorders by Kaslow et al. (2012) was a narrative rather than a systematic review. In addition, existing reviews have taken a problem focus and tended not to report outcomes related to positive development and the promotion of adolescent skills and competencies. Thus, to fully identify the role and scope of parenting interventions in addressing adolescent mental health problems from prevention to treatment, in‐depth and systematic exploration of the evaluation literature is required to understand the effectiveness of parenting interventions on adolescent mental health outcomes, positive development and the parent–adolescent relationship.

## 2 OBJECTIVES

The primary objective of this review is to answer the following research question: do parenting programmes for parents of adolescents impact adolescent mental health outcomes, positive development and the parent–adolescent relationship? We will achieve this by systematically searching for and synthesising the extant evaluation evidence that meets the inclusion criteria for this review. The secondary objective of this review is answer the research question: does the impact of parenting programmes for parents of adolescents on adolescent mental health outcomes, positive development, and the parent–adolescent relationship vary by: (a) diagnosis; (b) sociodemographic risk status (e.g., family structure, household income); (c) type of caregiver (e.g., biological parent, foster carer, kinship caregiver); (d) geographical location of the study; (e) intervention setting and modality; (f) type of outcome measurement modality (e.g., observation, self‐report); (g) type of intervention model (prevention, early intervention, treatment, theoretical framework); (h) participant age (parent and/or adolescent); and/or (i) participant gender (parent and/or adolescent)? Data permitting, we will fulfil this objective through subgroup analyses.

## 3 METHODS

### 3.1 Criteria for considering studies for this review

#### 3.1.1 Types of studies

This review will include randomised controlled trials (RCTs) where participants have been randomly allocated to an intervention or control condition, and cluster‐RCTs where predefined clusters or groups are randomised to different conditions. In these designs, the intervention condition refers to participants who take part in the parenting programme for parents of adolescents. The control group refers to those in a comparison condition involving no intervention, an alternative intervention, service provision or treatment as usual, or waitlist control. In addition, eligible studies may include follow up assessment.

#### 3.1.2 Types of participants

We define participation in a study to be comprised of two parts: (1) participation in the intervention and (2) the provision of outcome data. This means that participants can be either parents or adolescents. We envision that some studies might include adolescent data and not parent data, but to be included in the review the study must have one or more parent as a programme participant regardless of whether they provide outcome data.

Studies will be included if the participants are parents or caregivers who have adolescents aged between 10 and 18 years at the start of the intervention; accordingly, adolescent participants must be aged between 10 and 18 years. Parents or caregivers will be included if they are biological, adoptive, kinship, or foster caregivers. Adolescents may be the primary recipient of the intervention providing there is a substantive parenting component and that a key objective of the intervention is parenting support and capacity building. Where studies include a proportion of adolescents or parents with adolescents outside of the age range, we will contact the study authors to obtain data for participants in the specified age range. When that data is unavailable, we will include the study if at least 80% of the sample falls within the specific age range. If these data are not available, we will exclude the study. We will include studies in any resulting meta‐analyses regardless of whether the sample is comprised entirely of the eligible age group or comprised of at least 80% in the eligible age range. However, we will conduct sensitivity analyses to determine whether the results change by including studies with different proportions of eligible participants.

We will exclude parents with an uncontrolled serious mental illness (i.e., major depression, anxiety disorder, substance use disorder, bipolar disorder, psychotic disorder, personality disorder). These parents are arguably a unique category of participants and have been included in other reviews specifically targeted at these populations (Bee et al., 2014; Kersten‐Alvarez et al., 2010, 2011; Reupert et al., 2012; Siegenthaler et al., 2012; Thanhäuser et al., 2017). Adolescent participants will be excluded if they have a serious, uncontrolled mental illness or a diagnosis of intellectual disability, global developmental delay or traumatic brain injury. These individuals are likely to differ from neurotypically developing adolescents and the interventions would require a conceptually different and tailored approach that addresses the specific developmental issues and contexts of these individuals (Brown et al., 2013; Tellegen & Sanders, 2013).

#### 3.1.3 Types of interventions

Studies will be included that evaluate a parenting intervention that primarily targets parenting practices and/or the parent–adolescent relationship. The intervention can take a prevention, early intervention or treatment focus but must have an active psychological focus such as active skills teaching, CBT, acceptance or mindfulness, or emotion‐coping. We will include interventions based on any theoretical paradigm or approach, including parenting interventions based on behavioural principles and social learning theory, attachment theory (including Interpersonal therapy), family systems theories, relational frame theory/acceptance and commitment therapy and nonviolent resistance theory. Interventions involving an adolescent component (i.e., where the adolescent is a direct participant in the intervention) will be included only if the parenting component is primary, not supplementary, and is of equal or greater dosage than the adolescent component.

Interventions will be excluded if the sole focus or content of the intervention is related to the psychological functioning, social support, relationships or wellbeing of the parent (e.g., psychological interventions for mental health problems; case management or practical support for employment, housing and finances; social support groups, couples counselling). We will, however, include studies that have a parenting component alongside this type of content as long as the parenting aspect is of equal or greater dosage. We will exclude interventions that do not have an active psychological focus, such as those that involve peer support or psychoeducation only.

#### 3.1.4 Types of outcome measures

Studies will be included if the outcomes are measured using questionnaires (self or other report), independent observation, clinician ratings, administrative data or diagnostic interview. The timing of outcome assessment will be categorised as: short‐term (immediately postintervention to 3 months), medium‐term (3–6 months after the intervention) and long‐term (7–12 months vs. >12 months).

### 3.2 Primary outcomes

Studies with any of the following outcomes will be included in the review:

1. Adolescent mental health, including disruptive, oppositional or conduct behaviour problems; anxiety symptoms; and depressive symptoms; as measured by targeted, problem‐specific scales such as the Conners Rating Scales (Conners, 1997), Eyberg Child Behaviour Inventory (Eyberg & Ross, 1978), Child Depression Inventory (Finch et al., 1987), Screen for Child Anxiety Related Disorders (Birmaher et al., 1997), Spence Children's Anxiety Scale (Spence, 1998), and broad‐based questionnaires such as the Child Behaviour Checklist (Achenbach & Rescorla, 2001), Strengths and Difficulties Questionnaire (Goodman et al., 2003) and Youth Outcome Questionnaire (Burlingame et al., 2004).
2. Adolescent positive development (the skills and competencies that adolescents require to thrive and successfully transition into adulthood (Bowers et al., 2015; Lerner et al., 2009) including measures that assess social skills, educational achievement and engagement, persistence and dealing with setbacks, forward planning and preparedness, problem‐solving and optimism or hope for the future. This includes domain specific scales such as the Student Subjective Wellbeing Questionnaire (Renshaw et al., 2015), Social Skills Improvement System (Gresham & Elliott, 2008) or Children's Hope Scale (Snyder et al., 1997), as well as broad‐based measures assessing only positive development such as the Five Cs of Positive Youth Development (Geldhof et al., 2014) and those that include a subscale to measure positive development, such as the Adolescent Functioning Scale (Dittman et al., 2016) and Strength and Difficulties Questionnaire (Goodman et al., 2003).
3. Parent–adolescent relationship functioning or quality, including both positive (e.g., warmth, support, acceptance, connectedness) and negative (e.g., rejection, hostility, conflict) dimensions of the relationship and global measures of relationship quality.
4. Parenting practices, including effective (e.g., encouragement and praise, clear limit setting, appropriate consequences, consistency, appropriate monitoring) and ineffective (e.g., coercive or harsh discipline, physical discipline, neglect) dimensions of parenting and parenting styles.

### 3.3 Types of intervention settings

We will include studies with eligible interventions that are delivered in any setting (e.g., home, community, school, hospital, clinic, adolescent mental health services) or via any delivery modality (e.g., individual, group, workshop, seminar, online, telehealth). Included interventions can be of any duration or intensity, and may take a prevention, early intervention or treatment focus. Interventions can also be practitioner‐led or self‐directed (e.g., online programme, self‐help workbook). Finally, no restrictions will be placed on the geographical location of the intervention or the study. We recognise the impact that intervention setting and intensity may have on heterogeneity of effect sizes and so will conduct subgroup and sensitivity analyses to explore this heterogeneity (as specified in Section 3.5.8).

### 3.4 Search methods for identification of relevant studies

#### 3.4.1 Search strategy

Search terms were initially developed by examining similar reviews and eligible studies identified during scoping, following by identification of indexing terms in topic relevant databases. Moreover, a specialist librarian from the authors' academic institution was consulted. Search terms were tested and refined in consultation with the librarian and all authors. No restrictions will be placed on publication date or language. At the searching stage, we will only exclude clearly ineligible document types, if this option is available for the specific search location (e.g., book reviews, obituaries).

The search terms include the following categories: eligible population (adolescents), intervention type (parenting programme) and study design (RCT). Terms will be truncated where applicable (e.g., adol* to capture “adolescent,” “adolescents” or “adolescence”). Within the categories, search terms will be combined using the OR Boolean operator and NEAR/2 proximity operator (where available). Categories will then be combined using the AND Boolean operator. The search string will be applied to the title, abstract, keyword and index term fields, where possible, for all search locations. The exact structure of the search will be altered as required in accordance with the functionality of different searching platforms. Where the ideal advanced search cannot be implemented in a particular search location, a simplified version of the search strategy will be used. A search record will be used to record each search, per recommended guidelines (Kugley et al., 2017) and all searches will be reported in the final review.

The search terms are presented below in a generic format:

(Title OR Abstract OR Keywords OR Index Terms: (adol* OR teen* OR youth* OR

young* OR juven*))

AND

((Title OR Abstract OR Keywords: family* NEAR/2 (counselling OR education OR

intervention* OR program* OR therapy OR training OR treatment*)) OR (Title OR Abstract OR Keywords: (parent* OR mother* OR mum* OR mom* OR father* OR dad* OR filial OR caregiver* OR paternal OR maternal) NEAR/2 (course* OR class* OR counselling OR education OR intervention* OR program* OR therapy OR training OR treatment*)) OR (Title OR Abstract OR Keywords: "Family Intervention" OR "Family Therapy" OR "home intervention" OR "home program" OR "Parenting Training" OR "child‐parent Training" OR “parent management training” OR “parent training”))

AND

(Title OR Abstract OR Keywords: ("randomi* control* trial" OR RCT OR "randomi* clin* trial"))

#### 3.4.2 Electronic searches

To prevent publication bias, both published peer‐reviewed literature and unpublished grey literature will be included by searching key psychological electronic databases, trial registries and grey literature sources.

Databases

- MedLine
- Embase
- PsycINFO
- CINAHL
- ProQuest Platform: Dissertations and Theses Global, Social Science Databases, Social Services Abstracts
- Web of Science
- Cochrane and Campbell Collaboration libraries

Trial Registries

- Open Science Framework
- Cochrane Central Register of Controlled Trials (CENTRAL)
- Health Canada Clinical Trial Database
- Swiss National Clinical Trials Portal
- Philippine Health Research Registry
- South African National Clinical Trials Register
- Tanzania Clinical Trial Registry

Grey Literature sources

- International Bibliography of the Social Sciences
- PsycEXTRA
- Grey Literature Network Service
- Social Science Research Network
- Australian Research Alliance for Children and Youth
- Lifecourse Centre Working Paper Series
- OpenGrey
- Early Intervention Foundation
- Crime Solutions
- Australian Institute for Family Studies
- LILACS (Latin American and Caribbean Health Literature)
- World Health Organisation Institutional Repository for Information Sharing (WHO IRIS)
- Australian Institute for Health and Welfare (AIHW)
- National Technical Reports Library
- Australian Department of Health and Ageing Mental Health Publications
- BluePrints
- California Evidence‐Based Clearinghouse for Child Welfare

#### 3.4.3 Searching other resources

We will also conduct the following additional search strategies:

- Harvesting references from the reference lists from all included studies.
- Forward citation searching using GoogleScholar for all included studies. GoogleScholar is the chosen platform for citation searching, as its citation counts include studies that may not be published in peer‐reviewed journals.
- Hand searching the 12 months prior to the search date in topic relevant journals to identify any published articles that have not yet been indexed in databases. Richards (2008) demonstrated that hand searching is particularly valuable for identifying RCTs reported in abstracts, letters and languages other than English. The journals considered relevant for inclusion are: Australian Psychologist, Journal of Clinical Psychology, Clinical Child and Family Psychology Review, Child and Family Behavior Therapy, Journal of Child and Family Studies, Journal of Adolescence, Child and Adolescent Psychiatry and Mental Health, Child Psychiatry & Human Development, Journal of Family Psychology, Journal of Primary Prevention Youth Studies Australia, Journal of Adolescent Research, Prevention Science, Clinical Psychology Review, Academy of Child and Adolescent Psychiatry, Child Abuse and Neglect, Child Abuse Review, Journal of Adolescent Health, Journal of Child and Family Studies, Consulting in Clinical Psychology, Behaviour Research and Therapy, Lancet, Child Psychology and Psychiatry, Child: Care, Health and Development and Parenting: Science and Practice.
- Contacting the authors of included studies and parenting experts to identify any unpublished studies or studies not captured by the systematic search.

### 3.5 Data collection and analysis

#### 3.5.1 Description of methods used in primary research

Based on our current understanding of the extant literature, we expect eligible studies to include participants who are adolescents aged 10–18 at the commencement of the parenting intervention, and their parents. We expect participants will be recruited in primarily in community settings (e.g., schools), as well as social media and mental health organisations and that outcome measures will include questionnaires (self or other report), observation, clinician ratings, administrative data or diagnostic interview.

An example of a study that may be included is Yap et al. (2019). In this study, 332 adolescents aged between 12 and 15 years at the start of the intervention (*M* = 13.68 years; *SD* = 1.06 years) and their parents (*n* = 359) were randomly assigned to receive either the Partners in Parenting (PiP) program—a web‐based parenting intervention—or a control group who received five educational factsheets about adolescent development. Participants were recruited from secondary schools, online networks, social media and mental health organisations (e.g., Mental Health First Aid Australia). Outcomes included parent‐reported parenting, adolescent‐reported parenting and adolescent depression and anxiety symptoms. Specifically, the following primary outcome measures were used: The Parenting to Reduce Adolescent Depression and Anxiety Scale, Short Mood and Feelings Questionnaire, Spence Children's Anxiety Scale and the secondary outcome: The Parenting to Reduce Adolescent Depression and Anxiety Scale—Adolescent Report. These outcomes were measured at preintervention, postintervention and 12‐months follow‐up.

#### 3.5.2 Selection of studies

##### Title and abstract screening

The records captured by the systematic search will be exported to EndNote for the removal of clearly ineligible record types (e.g., book reviews, blog posts). The remaining records will be imported to SysReview (Higginson & Neville, 2014), where a two stage duplicate removal will be used. The first stage uses an automatic algorithm to remove duplicates based on the document title, secondary title (e.g., journal title), year of publication, and authors. SysReview retains the record from a duplicate pair that has a pre‐existing full‐text document within the EndNote library to expedite the full‐text literature retrieval stage following title and abstract screening. Potential duplicate records that are not exactly matched using the automated algorithm are then sorted by title to enable a manual duplicate removal process prior to title and abstract screening, whereby the review team will examine all the relevant citation fields to determine duplication.

All unique titles and abstracts remaining will undergo title and abstract screening using the following exclusion criteria:

1. Duplicate record;
2. Ineligible record type (e.g., book reviews, blog posts) and/or
3. The record does not include any topic related to parenting programmes for parents of adolescents.

Each record will be initially assessed to determine whether it is a duplicate or ineligible record type. While efforts will be made to exclude duplicate and ineligible document types prior to title and abstract screening, this step will act as a safeguard to prevent the presence of such records during full‐text screening. When records are confirmed to be unique and eligible record types, the third criterion will be applied to remove records that do not relate to parenting programmes for parents of adolescents.

Prior to independent screening, all review authors will independently and blindly double screen a test set of the same 25 titles and abstracts to ascertain inter‐rater reliability in screening decisions. The screening results for all review authors will be combined and assessed for consistency, with screening thresholds adjusted and more clearly documented to guide independent screening, where necessary. If required, a subsequent test‐set will be implemented to ensure screening decisions are consistent prior to independent screening. The remaining records will then be derived between review authors for independent screening. However, a random sample of 5% of exclusions from each screener will be cross‐checked and ensure screening consistency for the entire corpus of the search results. If the false negative rate exceeds 5%, additional samples of excluded records will be examined until the false negative rate falls below 5%.

Retained records will then progress to a full‐text review, preceded by a literature retrieval stage where the full‐text documents for potentially eligible studies are attached within SysReview to the relevant record. If full‐text versions cannot be located via existing university resources, they will be ordered via university libraries or by contacting document authors.

##### Full text eligibility screening

Records retained during the title and abstract screening stage will be screened using the following exclusion criteria:

1. Duplicate record;
2. Ineligible record type;
3. Document does not include topic related to parenting programmes for parents of adolescents;
4. Methodological design is not RCT;
5. Ineligible participants;
6. Ineligible intervention/no intervention and
7. Ineligible outcome measure(s).

The first three steps of the full‐text screening will be a final check to ensure that duplicates, ineligible record types and records which do not include topics related to parenting programmes for parents of adolescents have not proceeded to the full‐text screening stage.

Full text records will be independently screened by two review authors following the exclusion criteria. Any discrepancies in screening decisions will be mediated by a third review author or discussed with the entire review team if an unequivocal decision cannot be made by the third review author.

#### 3.5.3 Data extraction and management

Eligible documents progressing from the full‐text screening stage will be coded within *SysReview* using the coding companion provided in Appendix A. Broadly, the following information will be extracted and coded within the following domains:

1. General study characteristics (e.g., year published, authors, document type, country, funding source);
2. Participants (e.g., parent type and key demographic characteristics, adolescent key demographic characteristics, sample size and attrition, recruitment procedures);
3. Design and methods (e.g., study design, nature of control condition, unit of allocation, random assignment processes, group equivalence at baseline);
4. Intervention (e.g., name, aims and components, duration and dosage, setting, implementer or facilitator type, fidelity/integrity);
5. Outcomes measurement (e.g., name and type of measure, variable assessed, respondent and participant, time points, intervention effects);
6. Effect size data and
7. Risk of bias.

#### 3.5.4 Assessment of risk of bias in included studies

Two review authors will independently assess risk of bias for all included studies using the Cochrane Risk of Bias Tool 2.0 (Sterne et al., 2019). Specifically, studies will be rated as “high” or “low” risk of bias or “some concerns” across the following five domains: the randomisation process, deviations from intended interventions, missing outcome data, measurement of the outcome and selection of the reported result. While studies will be assessed for blinding of participants, we will place less emphasis on this domain as the participatory nature of interventions and the informed consent process underpinning the evaluation of the intervention makes blinding unrealistic. Where studies use a cluster randomised methodology, additional biases will be assessed, including: recruitment bias, baseline imbalances, loss of clusters and incorrect analyses (Higgins, et al., 2020).

#### 3.5.5 Measures of treatment effect

Given the nature of the existing research in this area, we anticipate that the treatment effects reported in the included studies will be predominantly continuous outcome measures. Subsequently, we expect to use standardised mean differences (SMDs) as the measure of treatment effect. Where studies report baseline and postintervention outcome data, SMDs will be calculated using postintervention outcomes. If eligible studies report binary outcomes, effect sizes will be computed as odds ratios, and then transformed into SMDs for meta‐analyses (Borenstein et al., 2009). For each outcome, we will convert the smallest number of effect sizes to a common effect size so that the final effect size metric denotes what is calculated most commonly across studies (Polanin & Snilstveit, 2016).

#### 3.5.6 Unit of analysis issues

The unit of analysis for this review will be each eligible study, rather than each report of a study. It is anticipated that some studies may be reported in multiple documents, may have multiple eligible outcomes, and/or multiple points of outcome measurement following the intervention. Additional unit of analysis issues relate to participant randomisation to clusters and multiple types of experimental or treatment groups within the one study. We will identify and consider all of these unit of analysis issues, as outlined below. The systematic review software used for this review allows for nested coding of studies (i.e., multiple outcomes within a study or multiple reports of a single study), which will facilitate taking these issues into account.

**Multiple study reports**

On occasion, studies may use the same sample of data across reports of a study or may use only a subset of a sample. All reports of an eligible study will be included in the review. However, we will only include one effect size for each conceptually distinct outcome in the meta‐analysis, using the most complete sample or portion of the sample that directly meets the inclusion criteria for the review (e.g., data for adolescents in a study sample, excluding data for children younger than the adolescent period). We will use all reports of a study to code and assess risk of bias and will state which study report is used for the calculation of effect sizes. In cases where the study characteristics or outcome data differs across study reports, we will contact the study authors to verify the information and will report both the variation in the reports and the information provided by the authors.

**Multiple conceptually analogous outcomes in one study**

Psychological research frequently reports multiple conceptually similar outcome measures within an individual study. If this is identified, we will code all study outcomes, but will combine conceptually equivalent outcomes into a composite effect size using the method described by Borenstein et al. (2009). This will mean only one effect size for each study is included in the meta‐analysis for each conceptually distinct outcome, which may entail a moderator analysis to examine whether the effects of the intervention vary by measurement modality (e.g., self‐report, other report).

**Multiple time points**

If studies report multiple outcome measurement time points, we plan to first categorise the postintervention measurements into short‐term (immediately postintervention to 3 months), medium‐term (3–6 months after the intervention) and long‐term (7–12 months and >12 months). Second, we will calculate effect sizes at each of these time points for each outcome category and then conduct separate meta‐analyses for each time point. We acknowledge that not all studies may equivocally fall into these time points and we may need to devise a cohesive categorisation that suits the studies located by the review.

**Cluster randomised trials**

Cluster randomised designs overestimate of the precision of study results (higher risk of Type I error) if the analysis conducted by the authors does not account for the dependence within clusters. If the review captures cluster‐randomised trials, they will first be assessed for the use of appropriate analyses to account for the clusters (e.g., multilevel modelling). If study authors analyse participants at the individual‐level and do not account for clustering, we will use the approach recommended by Higgins et al. (2020) to adjust the standard error prior to the study being included in a meta‐analysis.

**Multiple intervention groups and multiple interventions per individual**

Due to the nature of the intervention for this review, we do not anticipate locating studies where participants receive multiple interventions (e.g., cross‐over designs). If we locate studies where there are multiple intervention or treatment conditions, we will include and analyse the treatment condition that most closely aligns with the intervention inclusion criteria (Higgins, Li & Deeks, 2020).

#### 3.5.7 Dealing with missing data

The standardised coding form will identify where data is missing from a study (see Appendix A). Where missing data is identified, we will contact study authors to obtain the data. If after two contact attempts, the missing data cannot be obtained, the approach for dealing with the study will depend on the type of missing data. If the missing data is required for effect size calculation, the study will be excluded from meta‐analyses, but include in the qualitative summary of eligible studies. If the missing data is required for rating risk of bias, the missingness will be reported in the risk of bias summary tables and we will follow the direction of the risk of bias tool to arrive at the level of bias for the relevant domain. If the missing data relates to other characteristics of the study, we will comment on the overall missingness in the data when summarising the studies included in the review.

#### 3.5.8 Assessment of heterogeneity

We will use the *I* ^2^ statistic, *χ* ^2^ test and *τ* ^2^ (Higgins & Thompson, 2002) to assess the heterogeneity of each meta‐analysis. Should heterogeneity be identified, we will explore the sources of the heterogeneity using the methods outlined in Section 3.5.11.

#### 3.5.9 Assessment of reporting biases

Our comprehensive search strategy reduces the risk of reporting or publication biases. However, if there are sufficient studies, we will construct funnel plots and visually inspect for asymmetry. We will use subgroup analyses to ascertain whether the results of the meta‐analysis a significantly different depending on whether the included studies are published or unpublished documents.

#### 3.5.10 Data synthesis

We will enter all effect size data into RevMan (Version 5.4) and conduct random effects inverse variance meta‐analyses for each conceptually distinct outcome category where there is at least two independent studies. The results of the meta‐analyses will be presented in forest plots that also display the mean effect size and their corresponding 95% confidence intervals. All included studies, regardless of their inclusion in the meta‐analyses, will be summarised in narrative text and study summary tables according to key study characteristics (e.g., participations, intervention, outcomes, setting).

#### 3.5.11 Subgroup analysis and investigation of heterogeneity

If sufficient data is located, we will conduct subgroup analyses to examine whether the impact of the intervention changes according to the following variables: (a) diagnosis; (b) sociodemographic risk status (e.g., family structure, household income); (c) type of caregiver (e.g., biological parent, foster carer, kinship caregiver); (d) geographical location of the study; (e) intervention setting and modality; (f) type of outcome measurement modality (e.g., observation, self‐report); (g) type of intervention model (prevention, early intervention, treatment, theoretical framework); (h) participant age (parent and/or adolescent) and/or (i) participant gender (parent and/or adolescent). We will use meta‐regression to conduct subgroup analyses, in order to integrate multiple categorical and continuous moderators. If there are too few studies, which can result in overfitting (van Houwelingen et al., 2002), we will conduct moderator analysis analogous to analysis of variance. Additional exploratory subgroup analyses may be conducted depending on the studies located by the review, but we will explicitly distinguish between a priori and posthoc analyses in the final review. Subgroup analyses will be summarised in narrative text and tables.

#### 3.5.12 Sensitivity analysis

Provided there is enough data, we will conduct sensitivity analyses to ascertain the impact of different decision‐making processes on the results of the review. At protocol stage, we anticipate the following variables:

1. Level of risk of bias;
2. Different ICC estimates used when including cluster‐randomised trials;
3. Categorisation of the outcome measurement time points and
4. Percentage of study samples meeting inclusion criteria.

Specifically, we will rerun meta‐analyses with specific subgroups removed and examine the stability of the overall effect (e.g., removing studies with high risk of bias or <100% eligible participants). Additional exploratory sensitivity analyses may be conducted depending on the studies located by the review, but we will explicitly distinguish between a priori and posthoc analyses in the final review. For example, the categorisation of intervention models or outcome measures. Sensitivity analyses will be summarised in narrative text and tables.

#### 3.5.13 Treatment of qualitative research

We do not intend to include or synthesise qualitative research in this review.

## APPENDIX A FULL‐TEXT CODING FORM

**General Study Details**

1. Study ID [*textbox*]
2. Report ID [*textbox*]*
3. What type of document is this study? [*dropdown menu*]
  a. Peer‐reviewed journal article
  b. Book chapter
  c. Dissertation
  d. Conference presentation
  e. Government report, technical report, or working paper
  f. Other (specify in textbox)
4. How was this study located during the search process? [*dropdown menu*]
  a. Systematic search of electronic database
  b. Systematic search of non‐academic database
  c. Hand‐search or reference harvesting
  d. Professional contact
  e. Other (specify in textbox)
5. In what country was the intervention implemented? [*textbox*]
6. If the evaluation and/or intervention was funded, record the funding source. [*textbox*]

*SysReview allows for multiple reports of a single study to be included in the one full‐text coding record form. Each report is nested within the overall study record and the Report ID will consist of the Study ID followed by a unique alphabetical code (e.g., 1234_a, 1234_b…).

**Participants**

1. Who are the participants? [*checkboxes*]
  a. Parents (mothers only)
  b. Parents (fathers only)
  c. Parents (both mothers and fathers)
  d. Other caregiver (e.g., foster parents, grandparents)
  e. Adolescents (aged 10 to 18 years)
  f. Other (specify in textbox)
2. Describe the recruitment and sample for parent(s)/caregiver(s) using the fields below:
  a. How were participants recruited? [*textbox*]
  b. What were the eligibility criteria for inclusion in the study? [*textbox*]
  c. Describe the sample attrition. [*textboxes*]
    **Table:**
    Number of participants	Treatment	Comparison	Total
    Referred to study			
    Consented			
    Assigned			
    Began intervention			
    Completed intervention			
    Completed postintervention			
    Completed follow‐up 1			
    Completed follow‐up 2 (if applicable)			
  d. Describe the characteristics of the sample. [*textboxes*]
    **Table:**
    Characteristic	Treatment	Comparison	Total
    Age (mean, *SD*, range)			
    Gender (% female)			
    Ethnicity (proportions)			
    Socioeconomic status			
    Education			
  e. Note any other pertinent sample information (e.g., parity, marital status, parent mental health diagnosis or other key risk factors present). Please record for both the treatment and comparison groups [*textbox*]
3. If applicable, describe the recruitment and sample for children/adolescents using the fields below:
  a. How were participants recruited? [*textbox*]
  b. What were the eligibility criteria for inclusion in the study? [*textbox*]
  c. Describe the sample attrition. [*textboxes*]
    **Table:**
    Number of participants	Treatment	Comparison	Total
    Referred to study			
    Consented			
    Assigned			
    Began intervention			
    Completed intervention			
    Completed postintervention			
    Completed follow‐up 1			
    Completed follow‐up 2 (if applicable)			
  d. Describe the characteristics of the sample. [*textboxes*]
    **Table:**
    Characteristic	Treatment	Comparison	Total
    Age (mean, *SD*, range)			
    Gender (% female)			
    Ethnicity (proportions)			
    Mental health problem or diagnosis			
    Comorbidity			
  e. Note any other pertinent sample information (e.g., key risk factors present). [*textbox*]
4. How was adolescent mental health problem or diagnosis determined? [*textbox*]
5. What type of mental health problem/diagnosis was captured by the study? [*dropdown menu*]
  a. Externalising (e.g., disruptive, oppositional, conduct)
  b. Internalising (e.g., anxiety, depression)
  c. Both
  d. Other

**General Methodological Details and Nature of Comparisons**

1. What is the nature of the comparisons for this study?
  a. Single intervention contrasted with single comparison condition
  b. Multiple interventions against a single comparison condition
  c. Other (specify in textbox)
2. General research design classification [*dropdown menu*]
  a. Randomised controlled trial
  b. Cluster randomised controlled trial
  c. Other (specify in textbox)
3. What type of comparison condition was used? [*dropdown menu*]
  a. Waitlist control
  b. No treatment
  c. Service provision or treatment‐as‐usual (specify in textbox)
  d. Alternative treatment (specify in textbox)
  e. Other (specify in textbox)
4. What was the unit of allocation? [*dropdown menu*]
  a. Participant
  b. Dyads
  c. Family
  d. Service site
  e. Other (specify in textbox)
  f. Unclear
5. How were participants randomly allocated to conditions? [*dropdown menu*]
  a. Simple
  b. Yoked pairs
  c. Cluster (specify cluster in textbox)
  d. Block/stratified (specify variables in textbox)
  e. Matched pairs (specify matching variables in textbox)
  f. Other (specify in textbox)
  g. Unclear
  h. Not applicable
6. Who executed the randomisation? [*dropdown menu*]
  a. Researchers
  b. Practitioners
  c. Other (specify in textbox)
  d. Unclear
7. If applicable, was randomisation equivalent across intervention sites? [*dropdown menu*]
  a. Yes
  b. No
  c. Unclear
  d. No applicable
8. Was group equivalence assessed?
  a. Yes (specify how this was done in textbox)
  b. No
  c. Unclear
  d. Not applicable
9. Were the treatment and comparison groups equivalent at baseline?
  a. Yes
  b. No (specify differences in textbox)
  c. Unsure
  d. Not applicable
10. Are there any differences between participants who completed versus did not complete the treatment?
  a. Yes (specify differences in textbox)
  b. No
  c. Unsure
  d. Not applicable
11. What was the unit of analysis? [*dropdown menu*]
  a. Participant
  b. Dyads
  c. Family
  d. Service site
  e. Other (specify in textbox)
  f. Unclear

**Intervention Details**

1. What is the name of the intervention(s), as reported by study authors? [*textbox*]
2. When was the intervention conducted (e.g., year)? [*textbox*]
3. What settings were used during the intervention(s) (e.g., home, community, inpatient facility, school)? [*textbox*]
4. What delivery modality was the intervention provided to participants (e.g., individual, group, workshop, seminar, online, telehealth)?
5. Did the intervention include components other than the parenting component?
  a. Yes (specify other components in textbox)
  b. No
  c. Unsure
  d. Not applicable
6. Describe the intervention provided to participants. [*textbox*]
7. What is the focus of the intervention?
  a. Prevention
  b. Early intervention
  c. Treatment
  d. Other (specify in textbox)
8. Describe the duration of the **entire** intervention. If available, describe the minimum, maximum, mean and standard deviation for intervention duration. [*textbox*]
9. Describe the intensity of the **entire** intervention (e.g., frequency of contacts and length of contacts). If available, describe the minimum, maximum, mean and standard deviation for intervention intensity. [*textbox*]
10. If parenting is not the only component, describe the duration of the **parenting** component. If available, describe the minimum, maximum, mean and standard deviation for the parenting intervention duration. [textbox]
11. If parenting is not the only component, describe the intensity of the **parenting** component (e.g., frequency of contacts and length of contacts). If available, describe the minimum, maximum, mean and standard deviation for the parenting intervention intensity. [*textbox*]
12. Were adolescents included in the intervention?
  a. Yes (specify in what way and level of contact in textbox)
  b. No
  c. Unsure
  d. Not applicable
13. Who implemented the intervention? [*dropdown menu*]
  a. Nurse
  b. Social worker
  c. Psychologist
  d. Medical practitioner
  e. Other allied health practitioner
  f. Multiple practitioner types (specify in textbox)
  g. Unclear
  h. Other (specify in textbox)
14. Was there more than one intervention site? [*dropdown menu*]
  a. Yes (specify number of sites in textbox)
  b. No
  c. Unclear
15. Was treatment integrity monitored? [*dropdown menu*]
  a. Yes (specify in textbox)
  b. No
  c. Unclear
16. Were there any issues with fidelity? [*dropdown menu*]
  a. Yes (specify in textbox)
  b. No
  c. Unclear
17. Did the authors report cost‐benefit data? [*dropdown menu*]
  a. Yes (specify in textbox)
  b. No
  c. Unclear

**Outcome(s) Measurement***

*To be completed for each eligible outcome within a study (or group of reports for a study). To add another outcome, click the “Add another outcome” button located at the bottom of the screen.

1. What is the outcome being measured? [*textbox*]
2. What is the variable name that will be used in statistical software? [*textbox*]
3. Who does this outcome relate to? [*dropdown menu*]
  a. Parent/caregiver
  b. Adolescent
  c. Other (specify)
4. How was the outcome measured (e.g., name of scale)? [*textbox*]
5. What are the psychometric properties of the measurement tool (e.g., reliability, validity, diagnostic thresholds, what higher /lower values mean)? [*textbox*]
6. How was the outcome data gathered? [*dropdown menu*]
  a. Questionnaire
  b. Observation
  c. Professional report (e.g., mental health diagnosis)
  d. Official source (e.g., child protection status)
  e. Interview
  f. Other (specify in textbox)
7. Who was the respondent/participant? [*dropdown menu*]
  a. Adolescent
  b. Parent/caregiver
  c. Teacher
  d. Practitioner
  e. Other (specify in textbox)
8. At what time point(s) was the outcome measured? [*textbox*]
9. Were data collected in the same manner for the treatment and comparison conditions? [*dropdown menu*]
  a. Yes
  b. No (specify in textbox)
  c. Unclear
10. Which condition does the raw difference/effect favour (ignore statistical significance)? [*dropdown menu*]
  a. Experimental condition
  b. Comparison condition
  c. Neither condition (no difference)
  d. Unclear
11. In which direction did the outcome change? [*dropdown menu*]
  a. Positive
  b. Negative
  c. Mixed (specify in textbox)
  d. Unclear
12. Were there statistically significant differences for this outcome? [*dropdown menu*]
  a. Yes
  b. No
  c. Not tested
  d. Unclear
13. What were the study author(s)' conclusions about this outcome? [*textbox*]

**Effect Size Data***

*To be completed for each eligible outcome within a study (or group of reports for a study). To add another outcome, click the “Add another outcome” button located at the bottom of the screen.

1. On what page number is the effect size data reported? [*textbox*]
2. What type of effect size is being coded? [*dropdown menu*]
  a. Baseline or pre‐test measure prior to intervention
  b. Postintervention (first point of measurement after intervention)
  c. Follow‐up (subsequent point of measurement after first posttest)
3. What is the timeframe captured for the measure?
  a. Minimum [*textbox*]
  b. Maximum [*textbox*]
  c. Mean [*textbox*]
  d. Same for all participants (i.e., fixed) [*textbox*]
4. How was the effect size obtained for this outcome? [*dropdown menu*]
  a. Reported in document → *Go to Question 5*
  b. Calculated by user → *Go to Question 6*
5. Identify the type of effect size reported for this outcome and enter the required data for that effect size in the text boxes provided. [*textboxes*]
6. Enter the appropriate data in the relevant “Data for effect size calculations” tabs (see below). The data entered will depend on what is reported in the document. If none of the circumstances in the tabs reflect the data in the document, follow the link to David Wilson's online effect size calculator to calculate an effect size. You can enter the data in the “Data for effect size calculations 2” tab in the “Other information” textbox. [*textboxes*]
